# A case of giant juvenile fibroadenoma in an adolescent girl: a case report

**DOI:** 10.1186/s13256-026-05890-7

**Published:** 2026-03-12

**Authors:** Nagari Biratu Adugna, Gemechu Abera Negasa, Mathewos Tamene Terefe, Nagasa Biratu Adugna, Diriba Bekele Geleta

**Affiliations:** 1 Department of Surgery, Faculty of Medicine, Institute of Health, Jimma University, Jimma, Ethiopia; 2 Department of Pathology, Faculty of Medicine, Institute of Health, Wollega University, Nekemte, Ethiopia; 3 Department of Pediatric and Neonatal Nursing, Institute of Health Sciences, Wollega University, Nekemte, Ethiopia

**Keywords:** Giant juvenile fibroadenoma, Phyllodes tumors, Fibroepithelial lesions, Adolescent

## Abstract

**Background:**

Breast masses in adolescents are predominantly benign and are most commonly associated with fibroepithelial tumors. These tumors represent a heterogeneous group of biphasic lesions, ranging from benign fibroadenomatoid changes to malignant phyllodes tumors. Differentiating between fibroadenomas and phyllodes tumors is clinically significant owing to their differing malignant potential and the consequent implications for treatment strategies. However, this distinction is often challenging, as the two entities share considerable overlap in both clinical presentation and imaging characteristics. For this reason, surgical excision of the mass followed by histopathological evaluation remains essential to ensure accurate diagnosis and to prevent overlooking malignant forms with metastatic potential. Giant juvenile fibroadenoma, a rare subtype of fibroadenoma, accounts for approximately 0.5–4% of cases in adolescents. Although histologically benign, its characteristic rapid growth frequently necessitates surgical intervention to alleviate symptoms and exclude malignancy.

**Case presentation:**

We present the case of a 19‑year‑old female individual of Oromo ethnicity who developed a rapidly enlarging mass in the right breast. Surgical excision was performed, and histopathological evaluation confirmed the diagnosis of giant juvenile fibroadenoma.

**Clinical discussion:**

Giant juvenile fibroadenoma is an uncommon subtype of fibroadenoma, accounting for approximately 0.5–4% of cases in adolescents. It is defined by a tumor weight exceeding 500 g, a diameter greater than 5 cm, or a mass occupying more than four-fifths of the breast. Characteristically, it demonstrates rapid growth, leading to distortion and compression of the breast parenchyma. Although benign, its aggressive enlargement is particularly observed in children and adolescents. Diagnosis is primarily based on clinical evaluation and imaging studies. Clinically, the lesion presents as a well‑circumscribed, mobile mass with elastic consistency and clear contours, often resulting in breast deformity and aesthetic discomfort. Ultrasonography serves as the principal diagnostic modality, typically revealing a large, homogeneous, hypoechoic lesion. Given its rapid growth, surgical excision is the treatment of choice. Excision is warranted not only to prevent potential damage to the mammary gland but also to address cosmetic concerns and mitigate the negative impact on the patient’s quality of life.

**Conclusion:**

Although fibroadenomas, benign fibroepithelial lesions, represent the most common breast tumors in children and adolescents, histopathological confirmation remains indispensable to prevent misdiagnosis and to avoid overlooking malignant variants with metastatic potential.

## Introduction

Breast masses in adolescents are predominantly benign, with fibroepithelial tumors (FETs) representing the most common subtype. Malignant breast lesions in this age group are exceedingly rare, accounting for approximately 0.5% of cases [1]. Fibroepithelial lesions (FELs) of the breast comprise a heterogeneous group of biphasic tumors characterized by the concurrent proliferation of stromal (mesenchymal) and glandular epithelial–myoepithelial components [2]. These lesions encompass a broad histopathological spectrum, ranging from fibroadenomatoid changes at the benign end to malignant phyllodes tumors (PTs) at the aggressive end [3].

Fibroadenomas are benign fibroepithelial tumors and represent the most common breast lesions in children and adolescents. They account for approximately 30–50% of palpable breast masses during childhood and adolescence, and 44–94% of surgically excised breast masses within the same age group. A fibroadenoma is classified as giant when its diameter exceeds 5 cm, its weight surpasses 500 g, or when it replaces more than four-fifths of the breast tissue [4, 5]. When diagnosed in patients between 10 and 18 years of age, the lesion is designated as a juvenile fibroadenoma. A broader definition extends juvenile fibroadenoma to encompass all cases occurring during childhood and adolescence, up to 19 years of age, in accordance with the World Health Organization’s definition of adolescence [5, 6].

Giant juvenile fibroadenoma is a rare variant of fibroadenoma, accounting for approximately 0.5–4% of cases in adolescents. It is characterized by rapid growth and requires surgical confirmation following clinical and radiological evaluation. We report the case of a 19-year-old patient who presented with a rapidly enlarging mass in the right breast. The diagnosis of giant juvenile fibroadenoma was established clinically and subsequently confirmed by histopathological examination after surgical excision.

## Case presentation

A 19-year-old adolescent girl, of Oromo ethnicity, presented to the surgical outpatient department of Aira General Hospital, West Wollega, Ethiopia, on 18 August 2025 (Gregorian calendar), with a large right breast mass of 11 months’ duration. Over the preceding 4 months, the mass had progressively increased in size, causing significant concern for both the patient and her parents. She reported no breast pain, nipple discharge, myalgia, fever, dyspnea, cough, or loss of appetite. There was no history of breast trauma or recurrent breast lumps. The patient had no family history of breast or ovarian cancer and no known chronic illnesses. Menarche occurred at the age of 14 years, and her menstrual cycles have remained regular. She was unmarried and reported no history of sexual activity.

**Fig. 1:**
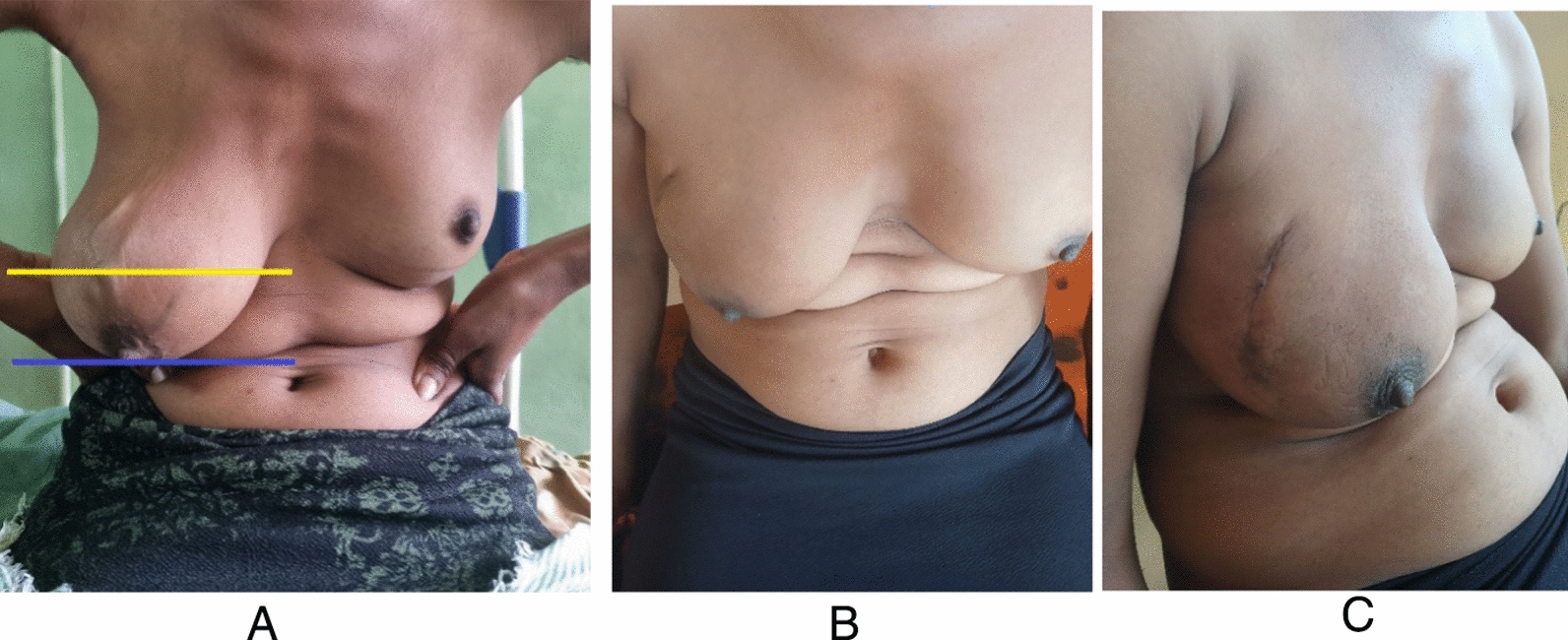
**A** Preoperative images showing a large right breast mass with severe ptosis. The yellow line indicates the level of the inframammary fold, while the blue line marks the position of the nipple. **B**, **C** Postoperative images following surgical excision

On physical examination, the patient’s body mass index (BMI) was 19.5 kg/m^2^, consistent with a lean body habitus. Marked breast asymmetry was observed, with the right breast significantly enlarged compared with the left. On the basis of the Regnault classification, the degree of breast ptosis was graded as severe. Figure 1A shows the presenting breast mass. The overlying skin appeared normal, with no evidence of discoloration or nipple discharge; however, dilated superficial veins were noted. Palpation revealed a firm, well-demarcated, mobile, nontender soft tissue mass measuring approximately 12 cm × 8 cm, occupying the entire outer quadrant of the right breast. No ipsilateral axillary lymphadenopathy was detected. Examination of the contralateral breast was unremarkable, and systemic evaluation revealed no additional abnormalities.

Ultrasound examination revealed a solid, well-demarcated hypoechoic mass occupying the majority of the outer quadrant of the right breast. The patient’s complete blood count was within normal limits. On the basis of the clinical presentation and sonographic findings, a diagnosis of giant juvenile fibroadenoma was considered. The patient was subsequently admitted to the surgical ward for further management.

**Fig. 2:**
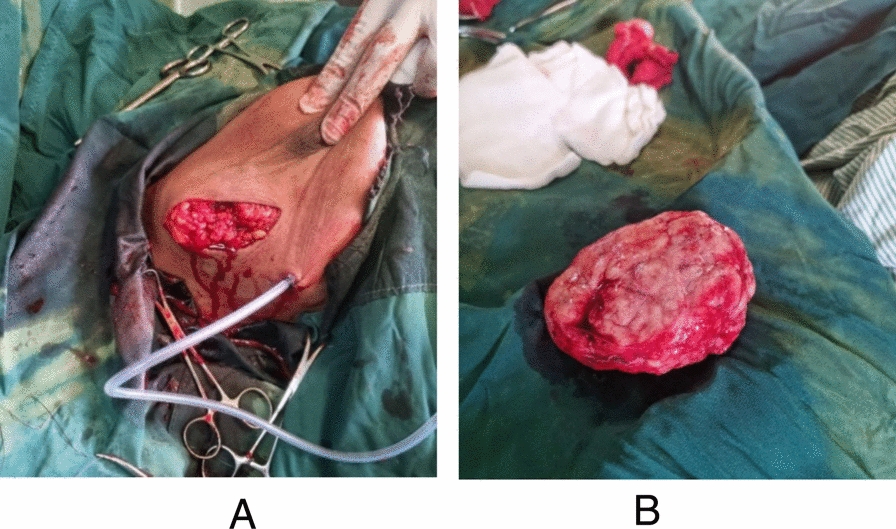
**A** Excision performed along the outer/lateral aspect of the right breast following removal of the giant mass. **B**, Gross appearance of the excised bulky tumor

The patient was thoroughly counseled regarding the nature of her giant breast mass, including the diagnosis, available treatment options, and associated risks and benefits. Following this discussion, she elected to undergo surgical excision. Written informed consent was obtained prior to the procedure. Under general anesthesia, a total surgical excision (enucleation) of the mass was performed along the outer envelope of the tumor, with careful preservation of the overlying skin to avoid unnecessary resection. An incision was made along the lateral aspect of the right breast (Fig. 2A). A bulky tumor measuring approximately 12 cm × 10 cm was successfully excised and submitted for histopathological evaluation (Fig. 2B).

The surgery achieved excellent cosmetic results, and the patient expressed high satisfaction with the outcome. Postoperative recovery was uneventful, and complete wound healing was observed at 4 weeks following surgery (Fig. 1C).

**Fig. 3:**
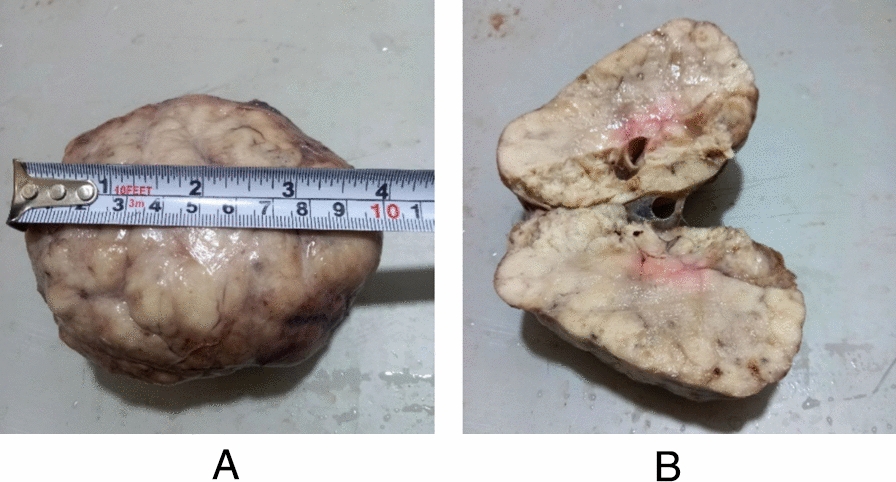
**A** Gross view of the excised right breast mass. **B** Cross-sectional view of the lesion demonstrating its lobulated internal architecture

Histological evaluation, including macroscopic examination, demonstrated a well-encapsulated mass with smooth margins. The cut surface revealed a solid, white–tan, lobulated appearance. The specimen measured 10 cm × 8 cm. Figure 3A depicts the gross view of the excised lesion, while Fig. 3B illustrates the cut section. Notably, the dimensions of the fibroadenoma differed from those estimated by ultrasound and immediate postoperative assessment, as histopathological measurement was performed 1 week later, by which time the excised mass had undergone shrinkage.

**Fig. 4:**
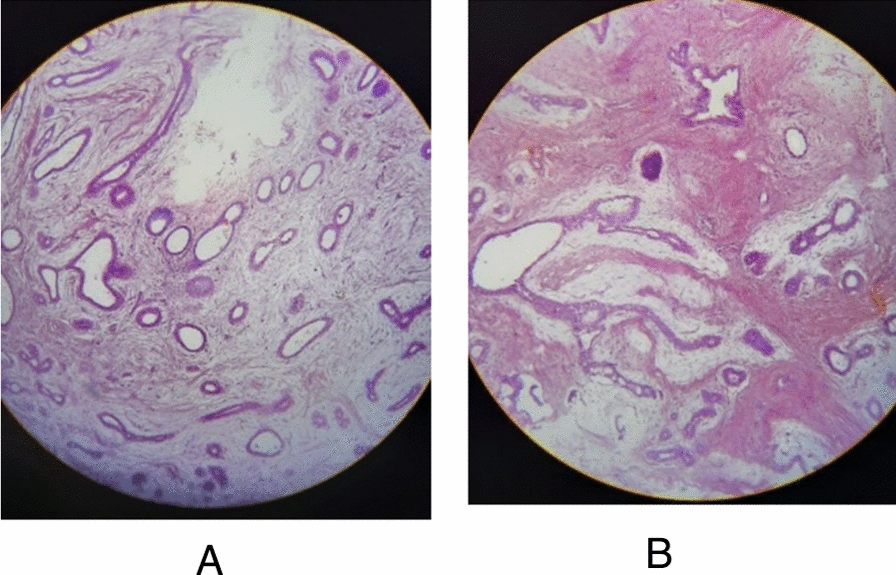
**A**, **B** Microscopic images of the excised right breast mass

Microscopic examination revealed numerous benign ducts lined by a double layer of epithelial and myoepithelial cells, without evidence of atypia. The background consisted of fibrous stroma, and no features suggestive of malignancy were identified. These findings confirmed the diagnosis of giant juvenile fibroadenoma. Figure 4A,B shows representative histological sections of the lesion.

## Discussion

The most common breast tumors in children and adolescents are fibroadenomas and their variants, which include fibroadenoid changes, simple fibroadenoma, complex fibroadenoma, cellular fibroadenoma, tubular fibroadenoma, lactating fibroadenoma, myxoid fibroadenoma, hyaline fibroadenoma, and giant juvenile fibroadenoma. Fibroadenomas are benign neoplasms composed of both glandular and stromal elements. They typically exhibit slow growth, except for hormonally sensitive subtypes such as juvenile fibroadenomas, lactating fibroadenomas, and phyllodes tumors[7].

Giant juvenile fibroadenoma represents a rare variant of fibroadenoma, accounting for approximately 0.5–4% of cases in adolescents. It is typically defined by a tumor weight exceeding 500 g, a diameter greater than 5 cm, or a mass occupying more than four-fifths of the breast. These lesions are characterized by rapid growth, often resulting in distortion and compression of the breast parenchyma [1, 5]. The condition predominantly occurs in children and adolescents. In a systematic literature review of 153 patients, Sosin *et al*. reported a mean age at diagnosis of 16.7 years, with an average lesion diameter of 11.2 cm [8]. One of the largest cases documented in sub-Saharan Africa was reported by Arowolo *et al*., involving a 14-year-old girl with a giant juvenile fibroadenoma measuring 20 cm × 30 cm in the left breast, which was surgically excised with synchronous breast reconstruction [9].

The precise etiology of juvenile fibroadenomas remains uncertain. Reproductive hormones are thought to contribute, as estrogen and progesterone receptors are expressed within fibroadenomas, and these lesions occur more frequently during puberty, pregnancy, and in individuals using oral contraceptives. A genetic predisposition may also play a role, given that fibroadenomas are reported with higher frequency among African American female individuals, and a positive family history of breast fibroadenomas is occasionally observed [10, 11].

In general, juvenile fibroadenomas are either minimally symptomatic or asymptomatic, typically painless, and most often detected incidentally during self-examination. They are usually unilateral and present as well-defined masses with smooth contours, elastic consistency, and mobility. In their giant form, these lesions may cause significant breast deformation, leading to aesthetic concerns and psychosocial distress [1, 12]. In the present case, the patient exhibited a right breast juvenile fibroadenoma measuring 12 cm × 8 cm, which had rapidly increased in size over a 4-month period, occupying nearly the entire breast and resulting in considerable discomfort and anxiety for both the patient and her parents. No family history of breast fibroadenoma was reported.

With regard to imaging, ultrasound remains the primary diagnostic modality. It typically demonstrates a large, homogeneous, hypoechoic mass that may exhibit hypervascularity on color Doppler and posterior acoustic enhancement. Such lesions tend to displace adjacent structures without evidence of invasion [7]. In the present case, ultrasound revealed a solid, homogeneous, well-demarcated hypoechoic mass without calcification or irregular margins, findings consistent with fibroadenoma.

In complex or hyalinized fibroadenomas, suspicious features may occasionally be observed, including irregular shape, noncircumscribed margins, or microcalcifications. In such circumstances, magnetic resonance imaging (MRI) can be performed, which generally demonstrates variable signal intensity, most often hyperintense on both T1- and T2-weighted sequences, with contrast enhancement that may be homogeneous or heterogeneous [7]. Mammography is not routinely performed in adolescents, given the low risk of breast malignancy and the radiographic density of adolescent breast tissue, which limits diagnostic utility [8]. When performed, mammography typically reveals a well-defined, oval-shaped opacity measuring greater than 5 cm [7].

The differential diagnosis between fibroadenomas and phyllodes tumors is clinically significant owing to differences in management strategies. Fibroadenomas may be safely observed or treated with simple enucleation, whereas phyllodes tumors require surgical excision with adequate margins to reduce the risk of local recurrence. Approximately 20–30% of phyllodes tumors are malignant, and nearly 25% of malignant phyllodes tumors metastasize [13, 14].

When suspicious features are present, distinguishing between fibroadenomas and phyllodes tumors can be challenging, as considerable overlap exists in their imaging characteristics. Consequently, definitive differentiation relies on complete surgical excision of the mass followed by histopathological evaluation [1, 13]. In the present case, ultrasound findings revealed no suspicious features suggestive of phyllodes tumor.

Histopathological examination of fibroadenomas is primarily characterized by abundant stromal proliferation accompanied by hyperplasia of the glandular epithelium, with peritubular patterns being more common than intratubular or mixed types [15]. The glands and interstitium are typically evenly distributed, and the extracellular matrix is predominantly composed of collagen. Necrosis and calcification are rarely observed. The ducts and lobules often demonstrate exuberant epithelial hyperplasia, which may assume grid-like, papillary, micropapillary, or solid architectural patterns. Nuclear features include overlapping nuclei, a water-like arrangement, and chromatin condensation. The interstitial component consists of bipolar spindle cells, with or without mild atypia, and mitotic figures are infrequent [16].

In rare instances, interstitial overgrowth may be present, raising concern for the concomitant development of phyllodes tumor structures[17]. In the present case, histological evaluation confirmed a benign giant fibroadenoma with no features suggestive of malignancy.

Many authors recommend that pediatric and adolescent patients with fibroadenomas demonstrating typical clinical and ultrasonographic features may be managed conservatively rather than surgically. Surgical excision carries potential risks, including scarring at the incision site, breast contour deformity, ductal system injury, and postsurgical mammographic changes that may reduce the diagnostic accuracy of mammography later in life. Furthermore, recurrence has been reported in approximately 10–25% of patients [18]. In conservatively managed cases, regular follow-up with ultrasound is considered a safe approach to confirm lesion stability over time. For patients seeking definitive diagnostic confirmation, fine-needle aspiration or core needle biopsy may be offered. However, if surgical excision has already been planned, preoperative core needle biopsy or other invasive diagnostic procedures should be avoided, as they may interfere with subsequent surgical management and cosmetic outcomes.

With regard to surgical management, the primary indications for excision of juvenile fibroadenomas include suspicious imaging characteristics or rapid tumor growth. Giant juvenile fibroadenomas are frequently treated surgically, as their rapid enlargement poses a risk of damage to the mammary gland, in addition to aesthetic concerns and impaired quality of life for the patient [8, 12]. In the present case, surgical resection was considered the optimal treatment owing to the rapid growth pattern of the mass and the patient’s preference for removal based on cosmetic considerations. Additional invasive procedures, such as core needle biopsy, were not performed, as they would not have altered the surgical plan.

At 4 weeks following excision of the giant mass, the patient demonstrated excellent postoperative recovery, with complete healing of the surgical site confirmed at follow-up (Fig. 1B,C). She expressed high satisfaction with the aesthetic outcome, noting that the procedure alleviated her physical discomfort and restored her confidence in her appearance. At the 3-month postoperative evaluation, no recurrence of the breast mass was detected on self-examination, a finding corroborated by the attending physician.

Giant juvenile fibroadenoma, although rare, should be considered in adolescents presenting with rapidly enlarging breast masses. Ultrasound serves as the primary diagnostic modality, while definitive differentiation from phyllodes tumor requires histopathological evaluation. Conservative management with imaging follow-up may be appropriate for typical fibroadenomas; however, surgical excision remains the treatment of choice in cases of rapid growth, aesthetic concern, or diagnostic uncertainty.

This case highlights that timely surgical intervention can achieve excellent cosmetic outcomes, alleviate patient anxiety, and prevent progressive breast distortion. At short-term follow-up, no recurrence was observed, underscoring the effectiveness of complete excision in managing giant juvenile fibroadenomas.

## Conclusion

Rapidly enlarging breast masses in children and adolescents can be a source of significant concern for both patients and their parents. Fibroadenomas, benign fibroepithelial lesions, represent the most common breast tumors in this age group; however, histopathological confirmation remains essential to avoid overlooking malignant variants with metastatic potential.

The management of breast disease in pediatric and adolescent populations differs from that in adults. Special surgical considerations must be directed toward preserving the developing breast parenchyma, minimizing injury to the ductal system, and maintaining future lactation potential. Equally important is achieving satisfactory cosmetic outcomes, given the young age of the patients and the psychosocial impact of breast deformity.

### Prognosis

Malignant transformation within a pre-existing juvenile fibroadenoma is exceedingly rare. Importantly, fibroadenomas are not associated with an increased risk of breast cancer in the general population [19, 20].

## Data Availability

The data supporting the findings of this study are available from the corresponding author upon reasonable request.

## Abbreviations

FET: Fibroepithelial tumors
FEL: Fibroepithelial lesions
PT: Phyllodes tumors
